# Bumble bee queens activate dopamine production and gene expression in nutritional signaling pathways in the brain

**DOI:** 10.1038/s41598-021-84992-2

**Published:** 2021-03-09

**Authors:** Ken Sasaki, Kakeru Yokoi, Kouhei Toga

**Affiliations:** 1 Graduate School of Agriculture, Honeybee Science Research Center, Tamagawa University, Machida, Tokyo, 194-8610 Japan; 2 Insect Genome Research Unit, Division of Applied Genetics, The National Agriculture and Research Organization, Institute of Agrobiological Sciences, Owashi 1-2, Tsukuba, Ibaraki 305-8634 Japan; 3 Department of Biosciences, College of Humanities and Sciences, Nihon University, Sakurajyosui 3-25-40, Setagaya-Ku, Tokyo, 156-8550 Japan

**Keywords:** Entomology, Social evolution, Insect hormones

## Abstract

To explore the neuroendocrine mechanisms underlying caste-specific behavior and its evolution from primitive to advanced eusocial bees, the monoamine levels and expression of genes involved in monoamine production and signaling in the brain were compared between the castes of *Bombus ignitus*. Higher levels of dopamine and its related substances were found in the brains of newly emerged queens than in the brains of emerged workers. The degree of caste differences in *B. ignitus* was smaller than that reported in *Apis mellifera*, indicating a link to different social stages in the two species. There was no differential expression in genes involved in dopamine biosynthesis between castes, suggesting that the high dopamine production in queens was not largely influenced by the expression of these genes at emergence, rather it might be influenced by tyrosine supply. Genome-wide analyses of gene expression by RNA-sequencing indicated that a greater number of genes involved in nutrition were actively expressed in the brains of newly emerged queens in comparison to the emerged workers. Some of the expression was confirmed by real-time quantitative PCR. The signaling pathways driven by the expression of these genes may be associated with dopamine signaling or the parallel activation of dopamine production.

## Introduction

Phenotypic plasticity is an adaptive response by gene expression to diverse environmental conditions. This response generates various phenotypes including external and internal morphology, body color, longevity, life history, and behavior^1,2^. In social insects, especially social Hymenoptera, females express different phenotypes with diverse reproductive potential on the basis of various cues including nutritional states^3,4^, mechanical signals in the nest^5^ and abiotic environmental cues^6,7^ at different developmental stages. Among them, nutrition during larval development has a great influence on female caste determination. A sufficient nutritional state during the larval stage leads to females with higher reproductive potential, such as queens, while an insufficient nutritional state leads to females with lower reproductive potential, such as workers^3,4^. In honey bees, royal jelly-containing sugars and royalactin activate particular signal cascades comprising nutritional-sensing genes, which increases the juvenile hormone titer in hemolymph and induces the morphological and behavioral development of a queen^8–10^. Additionally, a universal constituent of honey and pollen, *p*-coumaric acid in worker-jelly alters caste-associated gene expression and inhibits the development of a queen^11^. Thus, nutrition is an important factor affecting caste differentiation through multiple physiological pathways.

Eusociality is the most developed social organization and is categorized into two social stages: primitively and advanced^3^. In primitively eusocial hymenopterans, including bumble bees and paper wasps, there is no morphological differentiation between queens and workers except for body size, but a division of labor is seen in reproduction. In contrast, in advanced eusocial species, including honey bees, stingless bees, and ants, morphological and behavioral caste differentiations have evolved. The behavioral caste differentiation in adults is based on differences in the neurophysiological characteristics of the central nervous system. In advanced eusocial species, the differential expression of genes including insulin-signaling and vitellogenin in adult brains has been reported^12^. These different gene expressions are considered to generate caste-specific characteristics in the brain. In primitively eusocial species, different gene expression between castes in adult brains or heads has been investigated in bumble bees^13–16^ and paper wasps^17,18^. However, few studies investigating the caste differences of neurophysiology in the brain have been reported. Findings of the caste differences in the brain physiology of bumble bees are comparable with honey bees and other advanced eusocial species and contribute to an understanding of the evolution of caste-specific behavior.

Biogenic amines act on neurons in the central nervous system and peripheral tissues, and modulate behavior in vertebrates and invertebrates^19–21^. In solitary insects, one of the biogenic amines, dopamine, is involved in the control of sexual behavior^22^ and locomotion^23^ as well as fecundity^24^ and longevity^25^. In eusocial Hymenoptera, dopamine is associated with ovarian activation in reproductive females in the honey bee^26–28^, bumble bees^29,30^, a paper wasp^31,32^, and ants^33–35^. In honey bees, the caste differences of dopamine levels in the brains have been reported^36–38^. The dopamine levels in the brain were approximately four times higher in queens than in workers. Dopamine can enhance locomotor activity^39^, flight activity^40^, and aggression levels against rivals^41^ in virgin queens and therefore higher dopamine levels support queen-specific behavior. The caste differences of brain dopamine appear during the late pupal stage with different expression of enzyme genes involved in dopamine biosynthesis^38^. In primitively eusocial bees including bumble bees, however, there are no studies that compare the dopamine levels in the brain between castes. Since the social level in this group with undistinguished morphological castes is more primitive than that in honey bees, smaller differences in dopamine levels may occur in the brain between castes.

In the present study, we compared the brain levels of dopamine and its related substances between castes in a primitively eusocial bumble bee. We focused on the biosynthetic pathways of functional monoamines derived from amino acids, tyrosine, and tryptophan. We also investigated a wide range of gene expression in the brain to discover relationships between monoamine syntheses and particular signaling or substances in the brain.

## Results

### Morphological characteristics

Newly emerged females were defined as queens (gynes) and workers based on emergence at different colony growth stages and had no distinctive characteristics of external morphology except for body size. Thoracic width among females differed significantly between queens and workers and was significantly larger in queens than in workers (Mann–Whitney U test, z =  − 5.41, *P* < 0.0001, Table 1). Newly emerged females possessed inactivated ovaries with four ovarioles found constantly in their abdomen. Spermatheca diameter was significantly larger in queens than workers (z =  − 5.383, *P* < 0.0001, Table 1). Thus, the body size and internal morphology of the females that we collected could be distinguished as different castes.

**Table 1:** Caste differences in thoracic width and reproductive organs in *Bombus ignitus*.

Caste	Thoracic width (mm) Mean ± S.E	No. of ovarioles	Spermatheca diameter (mm) Mean ± S.E	No. of samples
	*U* test		*U* test	
**Colony 1**				
Queen	7.958 ± 0.081	4	0.308 ± 0.006	10
Worker	6.646 ± 0.160	4	0.249 ± 0.006	10
	z = − 3.780, *P* < 0.001		z = − 3.780, * P* < 0.001	
**Colony 2**				
Queen	7.804 ± 0.096	4	0.295 ± 0.003	10
Worker	6.421 ± 0.119	4	0.250 ± 0.004	10
	z = − 3.780, * P* < 0.001		z = − 3.780, * P* < 0.001	
**Total**				
Queen	7.881 ± 0.064	4	0.301 ± 0.004	20
Worker	6.533 ± 0.100	4	0.249 ± 0.003	20
	z = − 5.410, * P* < 0.0001		z = − 5.383, * P* < 0.0001	

### Caste differences in the brain levels of dopamine and its related substances

**Figure 1:**
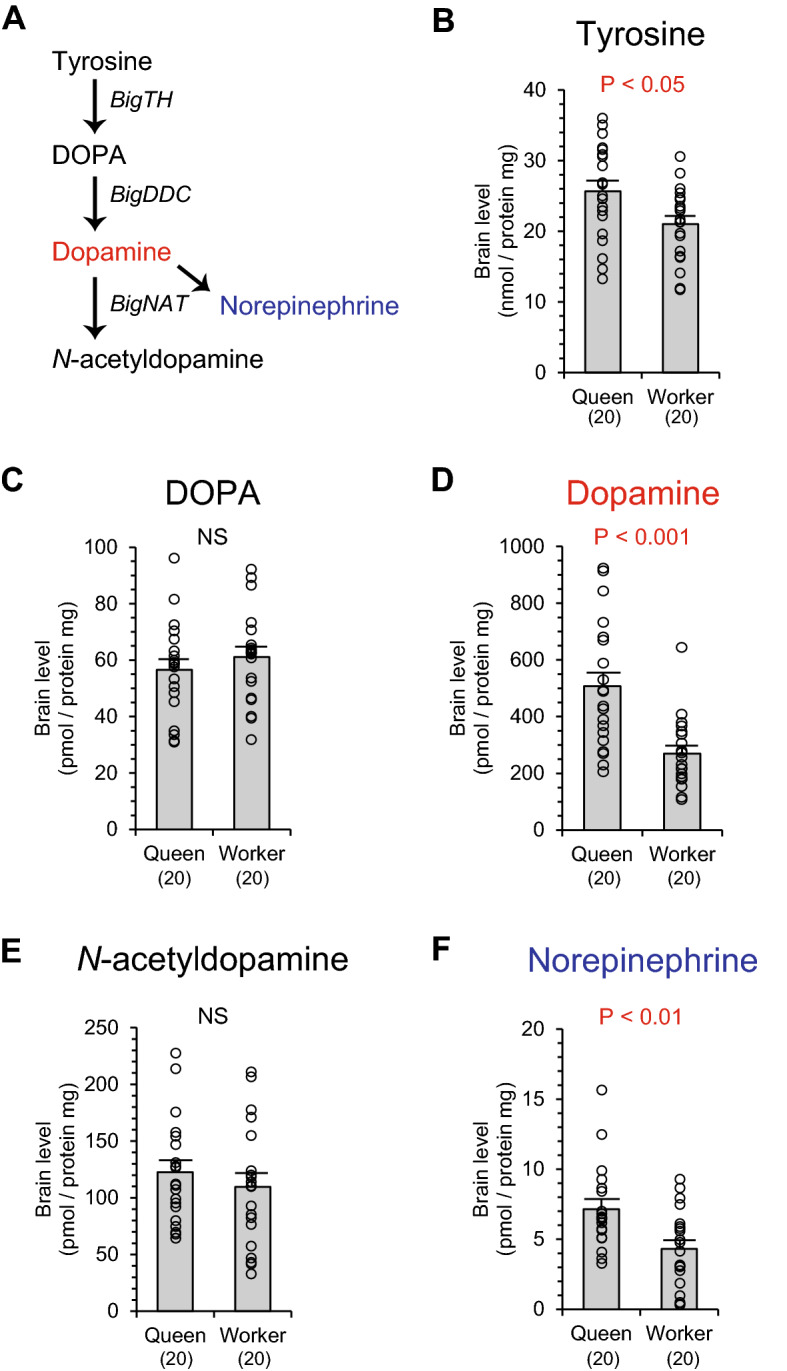
Levels of dopamine and its related substances in the brain of queens and workers in *Bombus ignitus*. Dopamine metabolic pathways are indicated (**A**). Dopamine is synthesized from tyrosine via DOPA and converted into *N*-acetyldopamine and norepinephrine. Enzyme genes mediating the biosynthetic reaction are indicated in italics. An enzyme gene converting norepinephrine has not been identified in insects. Dopamine is a functional monoamine (red) and norepinephrine is a potential functional monoamine (blue). Levels of tyrosine (**B**), DOPA (**C**), dopamine (**D**), *N*-acetyldopamine (**E**), and norepinephrine (**F**) in the brains of queens and workers are indicated. Numbers in parentheses indicate the number of samples examined. Data were obtained from 10 individuals in each caste per colony (two colonies). Individual data of biogenic amine levels were indicated by plots in graphs and in Table S7.

Dopamine is synthesized from tyrosine via DOPA and metabolized into *N*-acetyldopamine and norepinephrine (Fig. 1A). The amount of dopamine, its precursors, and metabolites were quantified in the brains and compared between newly emerged queens and workers (Fig. 1B–F). Levels of tyrosine, dopamine and norepinephrine in the brains were significantly higher in queens than workers (Mann–Whitney U test, tyrosine: z =  − 2.245, *P* < 0.05, Fig. 1B; dopamine: z = 3.706, *P* < 0.001, Fig. 1D; norepinephrine: z =  − 2.813, *P* < 0.01, Fig. 1F). In other dopamine related substances, DOPA and *N*-acetyldopamine, the brain levels did not significantly differ between castes (DOPA: z =  − 1.136, *P* = 0.256, Fig. 1C; *N*-acetyldopamine: z =  − 0.839, *P* = 0.402, Fig. 1E).

**Figure 2:**
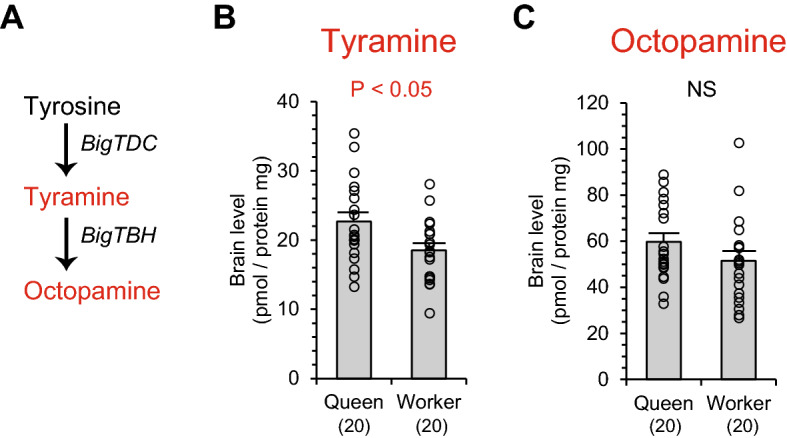
Levels of tyramine and octopamine in the brains of queens and workers in *Bombus ignitus*. A metabolic pathway of phenolamines is indicated (**A**). Octopamine is synthesized from tyrosine via tyramine. Enzyme genes mediating the biosynthetic reaction are indicated in italics. Tyramine and octopamine are functional monoamines (red). Levels of tyramine (**B**) and octopamine (**C**) in the brains of queens and workers are indicated. Numbers in parentheses indicate the number of samples examined. Data were obtained from 10 individuals in each caste per colony (two colonies). Individual data of biogenic amine levels were indicated by plots in graphs and in Table S7.

Tyrosine is not only a precursor of dopamine but also a precursor of tyramine and octopamine (Fig. 2A). Therefore, amounts of tyramine and octopamine in the brains were quantified and compared between castes (Fig. 2B,C). Levels of tyramine in the brains were significantly higher in queens than workers (Mann–Whitney U test, z =  − 2.056, *P* < 0.05, Fig. 2B). Levels of octopamine in the brains did not differ between castes (z =  − 1.461, *P* = 0.144, Fig. 2C).

**Figure 3:**
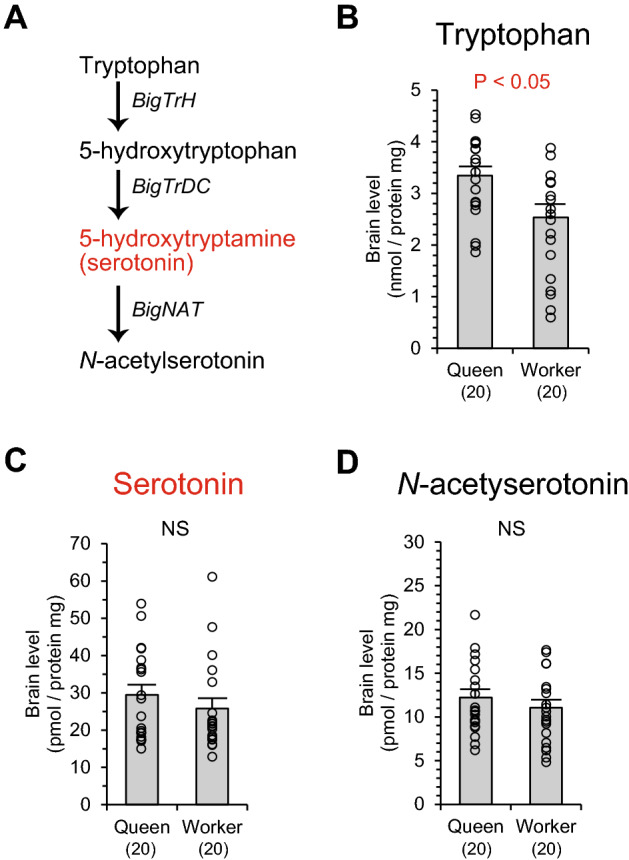
Levels of serotonin and its related substances in the brain of queens and workers in *Bombus ignitus*. A metabolic pathway of serotonin is indicated (**A**). Serotonin is synthesized from tryptophan via 5-hydroxytryptophan and converted into *N*-acetylserotonin. Enzyme genes mediating the biosynthetic reaction are indicated in italics. Serotonin is a functional monoamine (red). Amounts of tryptophan (**B**), serotonin (**C**), and *N*-acetylserotonin (**D**) in the brains of queens and workers are indicated. Numbers in parentheses indicate the number of samples examined. Data were obtained from 10 individuals in each caste per colony (two colonies). Individual data of biogenic amine levels were indicated by plots in graphs and in Table S7.

Serotonin is a functional monoamine on an independent synthetic pathway from dopamine, tyramine or octopamine, and derived from tryptophan (Fig. 3A). Levels of tryptophan in the brains were significantly higher in queens than workers (Mann–Whitney U test, z =  − 2.407, *P* < 0.05, Fig. 3B), whereas levels of serotonin did not differ between castes (z =  − 0.839, *P* = 0.402, Fig. 3C). This was true in a metabolite of serotonin, *N*-acetylserotonin. Levels of *N*-acetylserotonin did not differ between castes (z =  − 0.757, *P* = 0.449, Fig. 3D).

### Genome-wide analyses of gene expression by RNA-sequencing

**Figure 4:**
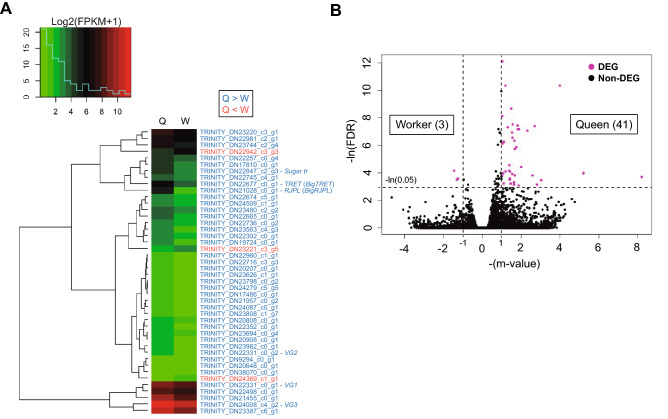
Differential gene expression between castes in *Bombus ignitus* was analyzed by RNA-seq. (**A**) a heat map of differential expression genes (DEGs) in queens (Q) and workers (W). The left panel above the graph indicates the frequency distribution of DEGs to log2(FPKM + 1). Expression levels increased from green to red. In the heat map, 41 DEGs were highly expressed in queens (blue text), whereas three DEGs were highly expressed in workers (red text). (**B**) a volcano plot of gene expression. Plots of DEGs and non-DEGs are indicated in magenta and black, respectively.

RNA-sequencing (RNA-seq) analysis was performed to compare various gene expression levels in the brains between the castes. Approximately 43–56 million raw reads were generated from cDNA libraries. Using the reads, we constructed contigs by de novo assembly. The basal statistics of the contigs are shown in Table S1. Forty-four differentially expressed genes (DEGs) were detected in a comparison between queens and workers (Fig. 4 and Table S2). Of these, significantly higher expression was detected in 41 DEGs in queens, whereas in workers three DEGs were detected (Fig. 4), indicating that a high expression was found in more numerous genes in queens than in workers. Thirty of 44 DEGs were functionally annotated by blastp and HUMMER search and defined locus names (Table S2). Six DEGs including a trehalose transporter gene (*TRET*), a sugar transporter gene (*Sugar tr*), a royal jelly protein-like gene (*RJPL*), and vitellogenin-like genes (*VG1*, *VG2*, and *VG3*) were nutrition-related genes (Fig. 4 and Table S3). We found no DEGs encoding enzymes involved in the metabolism of biogenic amines, receptors of biogenic amines, and a dopamine transporter (Table S4).

### Gene expression analyses by real-time quantitative PCR

**Figure 5:**
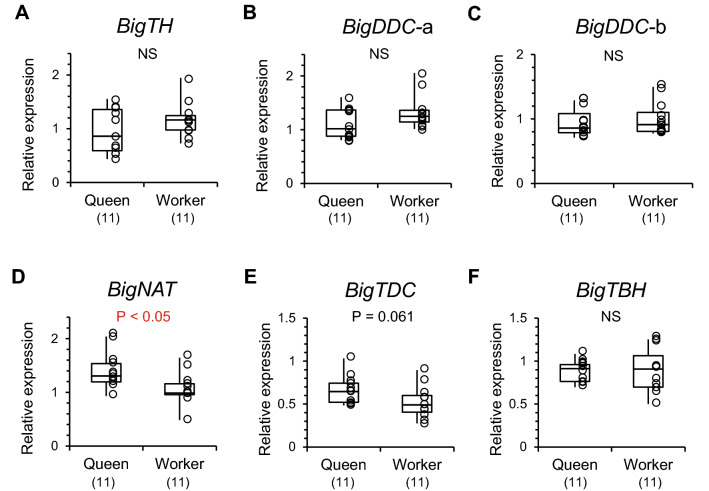
Relative expression levels of enzyme genes involved in the metabolism of dopamine, tyramine, and octopamine were analyzed by qPCR. (**A**) *Bombus ignitus* tyrosine hydroxylase gene (*BigTH*), (**B**,**C**) *B. ignitus* DOPA decarboxylase (*BigDDC*-a and *BigDDC*-b), (**D**) *B. ignitus N*-acetyltransferase (*BigNAT*), (**E**) *B. ignitus* tyrosine decarboxylase (*BigTDC*), F: *B. ignitus* tyramine-β-hydroxylase (*BigTBH*). Numbers in parentheses indicate the number of samples examined. Individual data of relative gene expression levels were indicated by plots in graphs and in Table S8.

To confirm and re-analyze the expression of specific genes in the brain, quantification of gene expression was carried out by real-time quantitative PCR (qPCR). In five genes related to tyrosine metabolism that we examined (Fig. 5), only *BigNAT* (*N*-acetyltransferase) expression levels were significantly higher in queens than in workers (Mann–Whitney U test, z =  − 2.265, *P* < 0.05, Fig. 5D). Expression levels of dopamine synthetic genes (*BigTH*, tyrosine hydroxylase, and *BigDDC*, DOPA decarboxylase), a tyramine synthetic gene (*BigTDC*, tyrosine decarboxylase), and an octopamine synthetic gene (*BigTBH*, tyramine β-hydroxylase) did not differ between castes (*BigTH*: z =  − 1.018, *P* = 0.309, Fig. 5A; *BigDDC*-a: z =  − 1.543, *P* = 0.123, Fig. 5B; *BigDDC*-b: z =  − 0.755, *P* = 0.450, Fig. 5C; *BigTDC*: z =  − 1.871, *P* = 0.061, Fig. 5E; *BigTBH*: z =  − 0.427, *P* = 0.670, Fig. 5F). Differences of the expression levels of *BigTDC* between castes were marginal (*P* = 0.061) and tended to be higher in queens than in workers (Fig. 5E).

**Figure 6:**
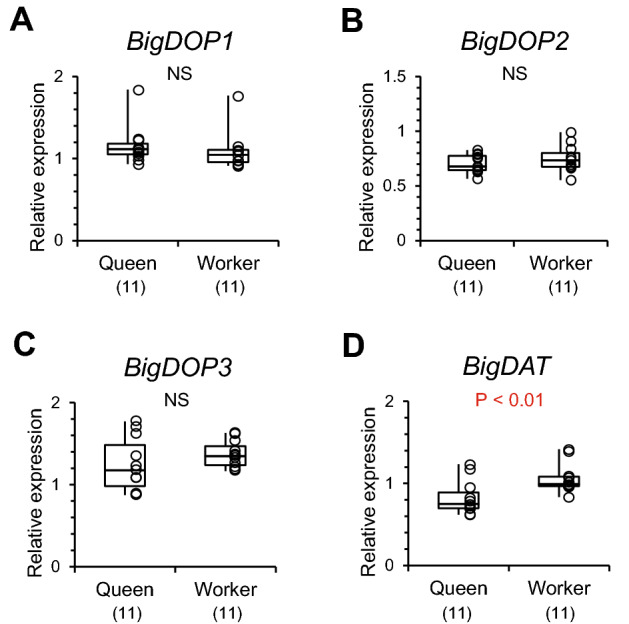
Relative expression levels of dopamine receptor genes and a dopamine transporter gene analyzed by qPCR. (**A**) *Bombus ignitus* dopamine receptor 1 gene (*BigDOP1*), (**B**) *B. ignitus* dopamine receptor 2 gene (*BigDOP2*), (**C**) *B. ignitus* dopamine receptor 3 gene (*BigDOP3*), (**D**) *B. ignitus* dopamine transporter gene (*BigDAT*). Numbers in parentheses indicate the number of samples examined. Individual data of relative gene expression levels were indicated by plots in graphs and in Table S8.

In four genes encoding dopamine receptors and a dopamine transporter, only *BigDAT* (dopamine transporter) expression levels were significantly higher in workers than in queens (Mann–Whitney U test, z =  − 2.725, *P* < 0.01, Fig. 6). Expression levels of dopamine receptor genes (*BigDOP1*, *BigDOP2*, and *BigDOP3*) did not differ between castes (*BigDOP1*: z =  − 1.412, *P* = 0.158, Fig. 6A; *BigDOP2*: z =  − 0.952, *P* = 0.341, Fig. 6B; *Big DOP3*: z =  − 1.280, *P* = 0.200, Fig. 6C).

**Figure 7:**
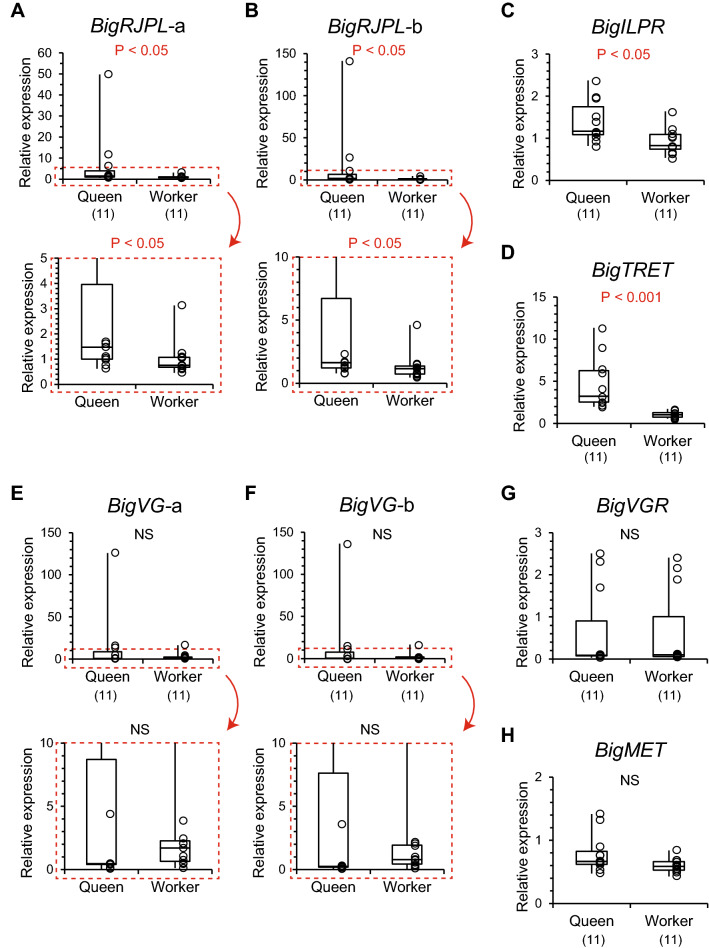
Relative expression levels in six genes involved in nutrition and a juvenile hormone receptor gene analyzed by qPCR. (**A**,**B**) *Bombus ignitus* royal jelly protein-like gene (*BigRJPL*-a and *BigRJPL*-b), (**C**) *B. ignitus* insulin-like peptide receptor gene (*BigILPR*), (**D**) *B. ignitus* trehalose transporter gene (*BigTRET*), (**E**,**F**) *B. ignitus* vitellogenin gene (*BigVG*-a and *BigVG*-b), (**G**) *B. ignitus* vitellogenin receptor gene (*BigVGR*), (**H**) *B. ignitus* methoprene tolerance gene (*BigMET*). Numbers in parentheses indicate the number of samples examined. Expression of *BigRJPL*-a*, BigRJPL*-b*, BigVG*-a, and *BigVG*-b are enlarged in boxes (red dashed border). Individual data of relative gene expression levels were indicated by plots in graphs and in Table S8.

In six genes related to nutrition and juvenile hormone, expression levels of a royal jelly protein-like gene (*BigRJPL*), an insulin-like peptide receptor gene (*BigILPR*), and a trehalose transporter gene (*BigTRET*) were significantly higher in queens than in workers (Mann–Whitney U test, *BigRJPL*-a: z =  − 2.068, *P* < 0.05, Fig. 7A; *BigRJPL*-b: z =  − 2.134, *P* < 0.05, Fig. 7B; *BigILPR*: z =  − 2.462, *P* < 0.05, Fig. 7C; *BigTRET*: z =  − 3.973, *P* < 0.001, Fig. 7D). Expression levels of a vitellogenin gene (*BigVG*) were largely fluctuated among individuals in queens, but did not differ significantly between castes (*BigVG*-a: z =  − 0.755, *P* = 0.450, Fig. 7E; *BigVG*-b: z =  − 0.821, *P* = 0.412, Fig. 7F). Expression levels of a vitellogenin receptor gene (*BigVGR*) and a juvenile hormone receptor gene (*BigMET*) did not differ between castes (*BigVGR*: z =  − 0.164, *P* = 0.870, Fig. 7G; *BigMET*: z =  − 1.674, *P* = 0.094, Fig. 7H).

## Discussion

Caste differentiation in social Hymenoptera is a phenotypic plasticity in response to diverse nutritional states during the larval stage. This characteristic generates the high performance of individual tasks for colony growth and high efficiency of reproduction in the colony. The investigation of the physiological differences between castes at the adult stage in a bumble bee has been conducted to understand the physiological mechanism underlying caste-specific behavior and social evolution from primitively to advanced eusocial bees. The present study has demonstrated that there were caste differences of monoamine production in the brain, especially large amounts of dopamine in virgin queens in the primitively eusocial bumble bee, *B. ignitus*. Genome-wide analyses of gene expression by RNA-seq indicated that a greater number of genes involved in nutrition were actively expressed in the brains of newly emerged queens in comparison to the emerged workers. Some of the expression was confirmed by qPCR. The signaling pathways driven by the expression of these genes may be associated with dopamine signaling or parallel activation with dopamine production.

### Synthetic pathways of monoamines in the brain

We quantified functional monoamines (dopamine, tyramine, octopamine, and serotonin) and their precursors, and compared their levels between castes. These monoamines are synthesized from the amino acids, tyrosine or tryptophan. The brain levels of these precursors were significantly different between castes, and higher in virgin queens than workers, suggesting that these levels might be derived from sufficient nutritional conditions in queens and could influence the brain levels of monoamines. In fact, the levels of dopamine, norepinephrine, and tyramine were significantly higher in queens than workers. However, the levels of serotonin and its metabolite *N*-acetylserotonin did not significantly differ between castes. These results suggest that the production of dopamine and tyramine were selectively enhanced.

The enhanced production of dopamine in virgin queens is conserved between the bumble bee (*B. ignitus*) and honey bee (*A. mellifera*). Interestingly, the caste differences of dopamine levels in the brain were relatively larger in the honey bee than the bumble bee. The differences in the honey bee were about four-fold^37^, whereas those in the bumble bee were about two-fold or less. This degree of caste differences in dopamine levels may be associated with the degree of morphological caste differentiation in the whole body; less morphological differentiation in the bumble bee. Thus, the caste differences of dopamine levels in the brains may be linked to social stages.

Tyramine is also synthesized from tyrosine as well as dopamine. Tyramine is a functional monoamine with receptors and is also a precursor of octopamine. The levels of tyramine were significantly higher in virgin queens than workers, but those of octopamine did not differ between them, suggesting that the higher levels of tyramine in queens did not influence octopamine biosynthesis and may be used for tyramine signaling. In the honey bee, there is no report indicating higher tyramine levels in virgin queens. Instead, the reproductive workers in queenless colonies have higher tyramine levels in the brains than normal workers^42^. The reproductive workers in the honey bee also had higher dopamine levels than normal workers^27^, suggesting a similarity in the neuroendocrine state with virgin queens in *B. ignitus*. In reproductive workers of the honey bee, tyrosine intake can enhance higher levels of dopamine and tyramine, and promote ovarian activation and inhibit foraging behavior^43^. It also has been reported that the application of tyramine in queenless workers could accelerate ovarian activation^44^ and the production of queen-like pheromone^45^. Thus, the neuroendocrine state and its roles in virgin queens in *B. ignitus* may be more similar to that in reproductive workers than in virgin queens in *A. mellifera*.

### Expression of genes involved in dopamine biosynthesis and signaling, and nutrition

From the results of RNA-seq, highly expressed genes were more numerous in queens (41 genes) than workers (three genes). Among these genes, we could not find any DEGs encoding enzymes involved in dopamine biosynthesis, dopamine receptors, or dopamine transporters. To confirm the results, we examined the expression of these genes in the brains of different individuals from different colonies by qPCR. We detected only a higher level of expression in *BigNAT* in queens and a higher level of expression in *BigDAT* in workers. The former encodes an enzyme that converts dopamine into *N*-acetyldopamine, the latter encodes a dopamine transporter. The expression of both genes did not explain the larger amounts of dopamine and tyramine in the virgin queens. Therefore, the larger amounts of dopamine and tyramine might be caused by a large amount of substrate tyrosine derived from sufficient nutrition during the larval stage, and not largely influenced by the expression of genes involved in monoamine syntheses at emergence. In *A. mellifera*, an additional food intake during the larval stage can increase levels of tyrosine and dopamine in the brains in artificially reared females^46^. Such a tyrosine intake from food may occur in queens in *B. ignitus* and cause the caste differences of monoamine levels in the brains.

Although there were no differences in gene expression of enzymes involved in syntheses of dopamine and tyramine between castes at emergence in *B. ignitus*, the expression of these genes during the pupal stage might be higher in queens, resulting in higher levels of dopamine and tyramine at emergence. In *A. mellifera*, however, the different expression of these enzyme genes between castes is more pronounced in newly emerged adults than in the pupae^38^. In *B. ignitus*, since the gene expression levels at emergence did not differ between castes, the levels during the pupal stage may be similar between castes. Comparison of the gene expression patterns between castes during the pupal stage of *B. ignitus* is required.

Expression of a gene *BigRJPL* encoding royal jelly protein was significantly higher in queens than workers in *B. ignitus* by both RNA-seq and qPCR. In *A. mellifera* and *B. terrestris*, the genes encoding the royal jelly protein express both hypopharyngeal glands and brains^47–49^. Although the royal jelly protein has a nutritive function in the honey bee, other functions have also been implicated in both species. In honey bee workers, some of the nine duplicated genes change the expression levels in the brain depending on age^50^ and under isolated conditions^51^. In both species, however, there have been no reports indicating different expression levels of these genes between castes. Since the origin of the royal jelly protein genes are considered to be *yellow* genes that bind and modify biogenic amines such as DOPA and dopamine^48,52,53^, the royal jelly protein gene in the brain might have another function involved in dopamine metabolism.

Higher expression levels of *BigILPR* encoding an insulin-like peptide receptor and *BigTRET*, encoding trehalose transporter in queens were detected by RNA-seq and qPCR. These genes are involved in sugar-sensing and sugar-transporting, respectively, and might respond to sufficient nutritional states from the larval to the adult stage. Insulin signaling has an important role in detecting rich nutritional conditions, it is directed toward queens during the larval stage and increases juvenile hormone titer in eusocial Hymenoptera^10^. In *B. terrestris* adult females, the expression of the *insulin growth factor 1* (*IGF1*) and *insulin-like peptide receptors* (*lnR2*) genes in the brain were higher in virgin queens (7 days old) than workers (9 days old), whereas those of *locust insulin-related peptide-like* (*LIRP*) and *insulin-like peptide receptor 1* (*lnR1*) did not differ between virgin queens and workers^15^. In the paper wasp *Polistes metricus*, the expression of the *insulin-like peptide 2* (*PmILP2*) and *insulin-like receptor 1* and *2* (*PmlnR1 and PmlnR2*) genes were higher in virgin queens than workers^17^. In several species of ants, a single gene *insulin-like peptide 2* (*ILP2*) is upregulated in the brains of reproductives^54^. The expression of *ILP2* in the brains is downregulated by the addition of larvae in the nest and upregulated by larva removal in clonal raider ants *Ooceraea biroi*^54^. Thus, the higher expression of insulin-like peptide signaling genes in the brains of reproductive females is seen in many species of Hymenoptera. This characteristic may be conserved in *B. ignitus*.

In *Drosophila* females, insulin-like peptide signaling is upstream of juvenile hormone and dopamine production^55^. The knockdown of the insulin-like peptide receptor gene (*dlnR*) resulted in a decrease in the levels of juvenile hormone and dopamine, whereas the treatment of adult flies with insulin caused an increase in juvenile hormone and dopamine^55^. In *B. ignitus*, *BigILPR* encodes an insulin-like peptide receptor that was expressed higher in virgin queens with higher dopamine levels than workers. The causal relationship between insulin-signaling and dopamine production remains to be determined.

Expression of the vitellogenin-like genes (*VG1*, *VG2,* and *VG3*) was significantly higher in queens than workers in results obtained from RNA-seq, whereas expression of two portions of *BigVG* (*BigVG*-a and *BigVG*-b) encoding vitellogenin did not differ between castes by qPCR. Since the sequence of *BigVG* was different from *VG1*, *VG2*, and *VG3*, the caste-dependent expression might be seen in only particular sequences of vitellogenin-like genes. Furthermore, there was a large variation in the levels of *BigVG* by qPCR in queens. It is expected that the queens need more vitellogenin for egg production than workers, but it is unclear when *B. ignitus* queens produce vitellogenin in the brain. In *A. mellifera*, the expression of vitellogenin genes (*VG*s) in the head was higher in queens than in workers and increased as queens aged^12^. A similar expression of *VG* between castes has been reported in the heads of paper wasp queens^17,18^. In *B. ignitus*, it is possible that *BigVG* expression increases in queens as they are aging, this remains to be determined.

## Conclusion

This study has demonstrated for the first time the caste differences of dopamine production in the brain of *B. ignitus*. The degree of differences in *B. ignitus* was smaller than that in *A. mellifera*, suggesting a link in social stages between the two bee species. From the results of RNA-seq and qPCR, there were no DEGs involved in dopamine biosynthesis between castes, suggesting that high dopamine production in queens was not largely influenced by the expression of these genes, rather by the substrates of enzymes involved in dopamine biosynthesis, especially tyrosine. Additionally, several genes involved in nutrition were expressed higher in the brains of queens. The signaling pathways driven by these genes might be associated with dopamine signaling or the parallel activation of dopamine production.

## Materials and methods

### Bumble bee colonies

Commercially reared bumble bee (*B. ignitus*) colonies were kept by the procedure described in Sasaki et al.^30^. Newly emerged workers were taken from developing colonies (during the late social phase) that had produced 40–50 workers, but no males or new queens (gynes). We selected similar-sized individuals among the newly emerged workers and excluded extremely small workers to decrease the variation in the body size of our sample. Newly emerged queens taken from the same colonies had grown mature and produced new queens, males, and workers (competition phase)^56^. Thus, we collected typical workers and queens based on periods of emergence at different colony growth stages. Both newly emerged workers and queens were sampled using liquid nitrogen. The heads of workers and queens were stored in liquid nitrogen before the measurement of biogenic amines or gene expression analyses. The thoraxes and abdomens were stored at − 80 °C for morphology measurements. Queens and workers were obtained from eight colonies: two colonies for the measurement of morphology and biogenic amines, three colonies for RNA-seq, and three colonies for qPCR.

### Measurements of morphological characteristics

To evaluate the differences in morphological characteristics between castes, we measured the thoracic width and the diameter of spermatheca in the abdomen. The number of ovarioles in the ovary was also counted. Female reproductive organs were dissected from the abdomen in 0.1 M phosphate buffer (pH 7.0) under a dissecting microscope and images were obtained by using a digital camera. Photographic images were analyzed with commercially available computer software (Photomeasure, Kenis, Osaka, Japan).

### Measurements of biogenic amines

Frozen brains were dissected and treated for the analyses by high-performance liquid chromatography with electrochemical detection (HPLC-ECD), following Sasaki et al.^38^. Two HPLC-ECD systems were used to measure the level of biogenic amines in the brain. One HPLC-ECD system developed by Matsuyama et al.^43^ was used for dopamine precursors (tyrosine and DOPA) and a dopamine metabolite (norepinephrine) and comprised a solvent delivery pump (EP-300, EICOM, Kyoto, Japan), a refrigerated automatic injector (AS-4550, JASCO, Tokyo, Japan), and a C18 reversed-phase column (250 mm × 4.6 mm id., 5-µm average particle size, MG120, Osaka Soda, Osaka, Japan) maintained at 35 °C. An electrochemical detector (ECD-300, EICOM) set at 0.84 V was used under 35 °C. The mobile phase contained 83 mM citric acid monohydrate, 17 mM sodium acetate, 13 µM 2Na-EDTA, 2.3 mM sodium-1-octanesulfonate. Into this solution, 7% methanol was added. The flow rate was kept constant at 0.7 mL/min. The measurements of dopamine, *N*-acetyldopamine, tyramine, octopamine, serotonin, tryptophan, and *N*-acetylserotonin were made using another HPLC-ECD system developed by Sasaki and Nagao^27^ and comprised a solvent delivery pump (PU-2080, JASCO), a refrigerated automatic injector (AS-2057, JASCO), and a C18 reversed-phase column (250 mm × 4.6 mm id., 5-µm average particle size, UG120, Osaka Soda) maintained at 35 °C. An electrochemical detector (ECD-700, EICOM) set at 0.85 V was used under 35 °C. The mobile phase contained 0.18 M monochloroacetic acid and 40 µM 2Na-EDTA, which was adjusted to pH 3.6 with NaOH. Into this solution, 1.62 mM sodium-1-octanesulfonate and 5% CH_3_CN were added. The flow rate was kept constant at 0.7 mL/min.

In both HPLC-ECD systems, external standards were run before and after the sample runs for the identification and quantification of functional monoamines (dopamine, tyramine, octopamine, and serotonin), dopamine precursors (DOPA and tyrosine), dopamine metabolites (*N*-acetyldopamine and norepinephrine), a serotonin precursor (tryptophan), and a serotonin metabolite (*N*-acetylserotonin). The peaks of these substances were identified by comparing both the retention time and the hydrodynamic voltammograms with those of the standard. Measurements based on the peak area of the chromatograms were obtained by calculating the ratio of the peak area of a substance to the peak area of the standards.

To normalize the brain levels of biogenic amines based on protein content, proteins were quantified by the Bradford method^57^. Precipitated protein pellets were neutralized with 50 µL 0.5 M NaOH following biogenic amine extraction. After the ultrasonic dissolution of the pellet for 15 min, the solution was diluted with 200 µL 0.1 M phosphate buffer (pH 7.0). As a standard solution, 5 mg bovine serum albumin was dissolved in 0.5 M NaOH with 0.1 M phosphate buffer (1:4, 5 mg/mL) and diluted with the same buffer 1/10, 1/20, 1/40, 1/80-fold. The samples and standards were reacted using protein assay dye reagent (500–0006, Bio-Rad, Hercules, CA, USA) in a 96 well-plate and mixed for 5 min. The absorbance of the mixtures was measured by a microplate reader (SH-1200Lab, Corona Electric, Ibaraki, Japan) with a 595-nm wavelength. Protein concentrations in the brains were determined from the calibration curve of the standard solutions.

### RNA extraction for RNA-seq

Frozen brains were dissected in ice-cold double-sterilized 0.1 M phosphate buffer (pH 7.0) on a Peltier cooling unit at approximately 4 °C covered with plastic paraffin film (Parafilm, Bemis Company, Chicago, IL, USA) under a dissecting microscope. Dissected brains, including the subesophageal ganglion, were homogenized with an electric homogenizer (T10 + S10N-5G, IKA Works, Staufen, Germany) in extraction buffer from an ISOGEN kit (NipponGene, Tokyo, Japan). Total RNA was extracted from two brains using an RNA isolation kit according to the manufacturer’s instructions. During RNA extraction, the RNA was treated with rDNase (RT Grade for Heat Stop, Nippongene) for 15 min to remove genomic DNA and then mixed with stop solution at 70 °C for 10 min. The quality and quantity of extracted RNA were determined at 230, 260, and 280 nm using a microvolume spectrophotometer (Nanodrop 2000, Thermo-Fisher Scientific, Waltham, MA, USA). Five RNA samples of queens and workers (two samples from each of two colonies and one sample from one colony) were examined.

### cDNA library preparation and sequencing

Before preparing cDNA libraries, the total RNA integrity was checked by using an Agilent 2100 Bioanalyzer (Agilent Technologies, Santa Clara, CA, USA) (Table S5). Using the TruSeq Standard mRNA LT Sample Prep Kit (Illumina, San Diego, CA, USA), a library was constructed from total RNA according to the manufacturer’s protocol. RNA-seq was performed on the RNA samples using Illumina Novaseq 6000 platform (Illumina) by Macrogen Japan (Kyoto, Japan). The raw sequence data were deposited in the DNA Data Bank of Japan (DDBJ) Sequence Read Archives (DRA) (accession numbers: DRR226102–DRR226111).

### Constructions of reference contigs

All the raw reads were filtered and adapter sequences were removed by Trimmomatic version 0.36^58^. Reference contigs were constructed by Trinity version 2.4.0^59^ using the trimmed sequence data. The reference contig data were deposited in the Transcriptome Shotgun Assembly (TSA) database (accession numbers ICPS01000001-ICPS01132255). A “Gene_id” was allocated to each contig by Trinity software. Converting between accession numbers and Gene_id can be performed by using deposited data in the DDBJ Genomic Expression Archive (GEA).

Methods of annotating each contig were described in Uchibori-Asano et al.^60^. Briefly, the contig sequence data were analyzed as queries using blastx (e-value < 1e^−3^) against the National Center for Biotechnology Information non-redundant (NCBI-nr) protein database and a top-hit description was adopted as an annotation for each contig. Also, using Transdecorder bundled with Trinity r20140717, a candidate coding site (CDS) was predicted for each contig, and additional annotations were added by using the predicted CDS data with the Pfam domain database by HMMER3^61^.

### Analysis of differentially expressed genes

Transcripts per million (TPM), mapped tag counts as “expected_count” (not normalized), and fragments per kilobase of transcript per million mapped reads (FPKM) number (normalized), were calculated for each contig by RSEM version 1.2.7 operated by “align_and_estimate_abundance.pl” in the Trinity package version Trinity r20140717^59,62^. The output files of “align_and_estimate_abundance.pl” of each sample including TPM, expected_count, and FPKM were deposited in DDBJ GEA (accession number: E-GEAD-356). For the detection of DEGs between castes, iDEGES/edgeR in the TCC package version1.8.2 was used with a false discovery rate (FDR) < 0.05 and fold changes of normalized tag count > 2 with “expected_count data”^63^. Scripts and command lines operating these core part of RNA-seq analysis in this study are shown in Sasaki et al.^64^.

### Measurements of relative expression levels of focal genes in the brain by qPCR

To confirm the different gene expression between castes obtained by RNA-seq, qPCR analyses were carried out on the focal genes. RNA extraction was conducted by the same RNA-seq procedure from two brains. For single-strand cDNA synthesis, DNase-treated RNA (500 ng) was transcribed using a high-capacity cDNA Reverse Transcription kit (Applied Biosystems, CA, USA) according to the manufacturer’s instructions. Negative control samples without reverse transcriptase were treated using the same procedure. Eleven RNA samples of queens and workers (five samples from one colony and three from each of two colonies) were examined.

Five genes encoding enzymes involved in dopamine biosynthesis (*BigTH* and *BigDDC*) or dopamine degradation (*BigNAT*), tyramine biosynthesis (*BigTDC*), and octopamine biosynthesis (*BibTBH*) were selected as target enzyme genes for qPCR analyses (Table S6). Three dopamine receptor genes (*BigDOP1*, *BigDOP2*, and *BigDOP3*) were selected as target receptor genes (Table S6). A dopamine transporter gene (*BigDAT*) was also selected as a target gene. Another six genes involved in nutrition, including a royal jelly protein-like gene (*BigRJPL*), an insulin-like peptide receptor gene (*BigILPR*), a trehalose transporter gene (*BigTRET*), a vitellogenin gene (*BigVG*) and a vitellogenin receptor gene (*BigVGR*), and a juvenile hormone receptor gene (methoprene tolerant: *BigMET*), were examined for the quantification of gene expression. In *BigDDC*, *BigRJPL*, and *BigVG,* two different primer sets were prepared on the same gene sequence at different sites (*BigDDC*: *BigDDC*-a and *BigDDC*-b; *BigRJPL*: *BigRJPL*-a and *BigRJPL*-b; *BigVG*: *BigVG*-a and *BigVG*-b). Since there were several sequences of vitellogenin-like genes, an identified sequence of a vitellogenin gene (*BigVG*) that was recorded in GenBank (accession number: FJ913883.1) was adopted for designing primer sets for *BigVG*. Three reference genes (actin 5C, *BigACT*; glyceraldehyde-3-phosphate dehydrogenase 2, *BigGAPDH*; and 40S ribosomal protein S3, *BigRPS3*) were examined with sets of primers (Table S6). The primer sequences of target and reference genes were designed using Primer 3 Plus (www.bioinformatics.nl/cgi-bin/primer3plus/primer3plus.cgi). Standard regression lines were generated for each target and reference gene (1, 1/5, 1/10, and 1/100 dilutions) and based on the relative concentration of cDNA and the quantification cycle (Cq). The cDNAs from newly emerged adult workers were used as a qPCR template. The qPCR was performed by the procedure described in Sasaki et al.^38^. An individual sample was repeated three times in a single run of the qPCR. The amplification of the single product was confirmed by dissociation curve analysis using a real-time PCR system.

To estimate the mRNA expression levels of each target gene, we recorded the Cq values of reference and target genes. The suitability of three reference genes as internal control genes (*BigACT*, *BigGAPDH*, and *BigRPS3*) was evaluated by a Mann–Whitney U test. The Cq values of *BigGAPDH* were the most stable and not significantly different between castes (Mann–Whitney U test, N_queen_ = N_worker_ = 11, *BigGAPDH*: z =  − 0.558, *P* = 0.577; *BigACT*: z =  − 1.083, *P* = 0.279; *BigRPS3*: z =  − 2.594, *P* < 0.01). Therefore, we normalized the expression levels of target genes by using expression levels of *BigGAPDH*. These analyses were performed with reference to the Minimum Information for Publication of Quantitative Real-Time PCR Experiments guidelines^65^.

### Statistics

The Mann–Whitney U test was used to analyze the morphological measurement and the levels of biogenic amines and the relative expression levels in each gene between castes.

### Ethic approval

This article does not contain any studies with human participants. All applicable international, national, and/or institutional guidelines for the care and use of animals were followed.

## Supplementary Information

Supplementary Tables.

## Data Availability

The raw RNA-sequence data were deposited in the DNA Data Bank of Japan (DDBJ) Sequence Read Archives (DRA) (accession numbers: DRR226102- DRR226111). The reference contig data were deposited in the Transcriptome Shotgun Assembly (TSA) database (accession numbers ICPS01000001-ICPS01132255). The output files of “align_and_estimate_abundance.pl” of each sample including TPM, expected_count, and FPKM were deposited in DDBJ GEA (accession number: E-GEAD-356). Other data are within the paper and its Supplementary materials.
