# Supplementary material for: Bumble bee queens activate dopamine production and gene expression in nutritional signaling pathways in the brain
Source: Sci Rep. 2021 Mar 9;11:5526. doi: 10.1038/s41598-021-84992-2 (PMC7943803; doi:10.1038/s41598-021-84992-2)
Supplement: Supplementary file 1 — Supplementary Tables. [file 41598_2021_84992_MOESM1_ESM.pdf]

Supplementary materials

Title: Bumble bee queens activate dopamine production and gene expression in nutritional signaling pathways in the brain.

Authors: Ken Sasaki, Kakeru Yokoi and Kouhei Toga

Table S1. RNA-seq contig statistics

Table S2. Different expression genes (DEGs) between castes in the brains in *Bombus ignitus*

Table S3. Identified different expression genes (DEGs) involved in nutrition between castes in the brains in *Bombus ignitus*.

Table S4. Expression of genes coding enzymes involved in monoamine metabolisms and monoamine receptors in queens and workers in the brains in *Bombus ignitus*.

Table S5. QC results of total RNA for RNA-seq libraries.

Table S6. Primer sequence of the focal genes for qPCR.

Table S7. Data of biogenic amine levels (pmol/protein mg) analyzed by HPLC-ECD.

Table S8. Data of relative gene expression levels analyzed by qPCR.

Table S1. RNA-seq contig statistics

Total number of contigs	132255
Median contig length	977
Total assembled bases	256567584
Percent GC	38.05
Contig N50	3948

These statistics were calculated by TrinityStats.pl in Trinity packages.

Table S2. Different expression genes (DEGs) between castes in the brains in *Bombus ignitus*.

Gene id	Top-hit ID (NCBI-nr)	Description (NCBI-nr)	Description (Pfam)	A-value	M-value	FDR	Q 1 (tag count)	Q 2 (tag count)	Q 3 (tag count)	Q 4 (tag count)	Q 5 (tag count)	W 1 (tag count)	W 2 (tag count)	W 3 (tag count)	W 4 (tag count)	W 5 (tag count)
TRINITY_DN22331_c0_g1	XP_003489778.1	PREDICTED: uncharacterized protein LOC100745498 isoform X2 [Bombus impatiens]	Vitellogenin_N(PF01347): VG1	14.15	-1.15	3.8E-06	32574	27277	19007	29173	27301	10194	10076	13682	12417	14513
TRINITY_DN22847_c2_g3	XP_003398210.1	solute carrier family 22 member 21 isoform X2 [Bombus	Sugar_tr(PF00083): Sugar tr	10.18	-1.03	5.5E-06	1674	1605	1547	2038	1436	888	914	821	727	723
TRINITY_DN22331_c0_g2	XP_012135210.1	PREDICTED: uncharacterized protein LOC100881008 isoform X1 [Megachile rotundata]	Vitellogenin_N(PF01347): VG2	4.16	-3.99	3.2E-05	109	17	7	131	91	2	5	6	3	4
TRINITY_DN22498_c0_g1	XP_003393207.1	uncharacterized protein LOC100645788 isoform X1 [Bombus terrestris]		14.22	-1.19	3.2E-05	33407	31126	24890	35346	19076	9742	9698	15430	14016	13976
TRINITY_DN22257_c0_g4	XP_003398056.1	DNA-directed RNA polymerase II subunit RPB1 [Bombus	MHCassoc_trimer(PF08831)	9.65	-1.49	0.00017	1025	2222	1385	1406	713	421	377	597	482	523
TRINITY_DN24008_c4_g2	XP_012175638.2	LOW QUALITY PROTEIN: uncharacterized protein LOC105667095, partial [Bombus terrestris]	Vitellogenin_N(PF01347): VG3	18.35	-1.02	0.0003	461675	734701	397809	403123	391357	234014	237586	256902	207103	239258
TRINITY_DN23694_c0_g4	XP_003492897.2	PREDICTED: dynein heavy chain 17, axonemal-like [Bombus impatiens]		4.76	-1.57	0.00054	60	34	45	55	36	24	13	7	15	17
TRINITY_DN17810_c0_g1			LRR_8(PF13855)	7.85	-1.82	0.00059	428	425	222	859	223	114	83	149	141	123
TRINITY_DN22665_c0_g1	XP_003400602.1	apyrase [Bombus terrestris]	Metallophos(PF00149),5_nucleotid_C(PF02872)	7.19	-2.7	0.00062	87	639	276	757	100	69	56	39	73	48
TRINITY_DN22745_c4_g1	XP_003494581.2	PREDICTED: LOW QUALITY PROTEIN: neurexin-1-like, partial [Bombus impatiens]		8.11	-1.32	0.00067	366	652	479	373	299	180	250	171	128	141
TRINITY_DN21957_c0_g2	No hits			4.72	-1.9	0.00077	34	98	53	42	25	15	10	14	13	13
TRINITY_DN24087_c0_g1	XP_020723306.1	uncharacterized protein LOC110120091 [Bombus terrestris]		4.2	-1.83	0.00078	25	30	57	24	34	7	6	13	5	14
TRINITY_DN22677_c0_g1	XP_003401245.1	facilitated trehalose transporter Tret1 [Bombus terrestris]	Sugar_tr(PF00083): TRET	10.85	-2.32	0.00086	3099	6403	4094	5590	1376	303	283	1214	1499	818
TRINITY_DN22960_c1_g1	No hits			4.47	-1.53	0.00098	40	51	20	40	33	12	11	15	15	10
TRINITY_DN21455_c0_g1	XP_012172148.1	uncharacterized protein LOC100646603 isoform X1 [Bombus terrestris]	Gp58(PF07902)	14.64	-1.55	0.00143	23859	91761	47547	32127	23080	12928	12003	15883	18014	15587
TRINITY_DN20808_c0_g1	XP_003401503.2	uncharacterized protein LOC100646043 [Bombus terrestris]	7tm_6(PF02949)	5.71	-1.66	0.00178	67	194	61	74	66	35	27	26	31	25
TRINITY_DN22981_c2_g1	ACA04898.1	apidaecin [Bombus ignitus]	Apidaecin(PF00807)	9.19	-1.12	0.00193	992	1052	838	932	476	478	293	444	371	391
TRINITY_DN22674_c5_g1	XP_020720715.1	LOW QUALITY PROTEIN: uncharacterized protein LOC100642575 [Bombus terrestris]		5.93	-1.03	0.0022	74	106	74	99	80	37	38	38	42	56
TRINITY_DN23798_c0_g2	XP_012167581.1	retinol dehydrogenase 10-A isoform X1 [Bombus terrestris]	[m.5379]:OTU(PF02338) | [m.5377]:adh_short(PF00106)	4.48	-1.76	0.00283	33	57	45	53	15	11	9	7	15	16
TRINITY_DN20908_c0_g1	No hits			4.33	-1.68	0.00324	48	41	16	44	30	6	8	13	10	16
TRINITY_DN23563_c4_g3	No hits			4.52	-1.39	0.0105	25	51	45	36	26	9	12	11	18	19
TRINITY_DN20207_c0_g1	No hits			4.16	-2	0.0121	12	61	57	30	16	9	5	11	9	9
TRINITY_DN24369_c1_g1	No hits			4.92	1.45	0.0158	26	23	11	11	18	40	25	51	68	63
TRINITY_DN23220_c3_g1	XP_003402013.1	ejaculatory bulb-specific protein 3 isoform X1 [Bombus	OS-D(PF03392)	11.75	-1.39	0.0158	4656	5622	5714	8907	3008	1781	814	2658	2979	2450
TRINITY_DN22302_c0_g1	No hits			7.43	-1.2	0.0165	187	483	253	205	177	111	113	111	102	129
TRINITY_DN23387_c6_g1	XP_012172141.1	uncharacterized protein LOC100643862 [Bombus terrestris]	Cyclin_N(PF00134),Cyclin_C(PF02984)	17.03	-1.43	0.0167	127808	375639	265047	234505	97397	59843	43730	90774	120047	95177
TRINITY_DN21028_c0_g1	ADV72544.1	major royal jelly protein [Bombus ignitus]	MRJP(PF03022): RJPL	7.71	-5.21	0.0188	24	4809	68	1421	51	109	6	20	29	5
TRINITY_DN23480_c2_g2	XP_020721170.1	uncharacterized protein LOC110119691 isoform X2 [Bombus terrestris]		8.41	-1.08	0.0188	442	791	539	396	294	189	216	243	266	253
TRINITY_DN9294_c0_g1	XP_003400053.1	uncharacterized protein LOC100648224 [Bombus terrestris]		4.05	-1.54	0.0201	23	30	30	42	15	5	9	16	7	9
TRINITY_DN23744_c2_g4	XP_012249947.1	PREDICTED: protein lethal(2)essential for life-like, partial [Bombus impatiens]		11.19	-1.67	0.0221	2010	10217	4049	2956	1600	1042	1074	1457	1758	1193
TRINITY_DN22352_c0_g1	ACA04899.1	hymenoptaecin [Bombus ignitus]		4.08	-2.59	0.0221	96	21	11	65	13	5	3	13	4	7
TRINITY_DN22736_c0_g2	XP_012164384.1	uncharacterized protein LOC100645593 [Bombus terrestris]		7.52	-1.02	0.0229	252	311	244	324	171	120	85	112	155	171
TRINITY_DN23982_c0_g1	BAG46934.1	hypothetical protein BMULJ_05092 [Burkholderia multivorans ATCC 17616]		1.67	-8.21	0.0248	10	1	0	0	262	0	0	0	0	0
TRINITY_DN17486_c0_g1	No hits			4.01	-1.42	0.0276	27	40	29	18	16	9	8	8	13	9
TRINITY_DN22942_c3_g3	No hits			6.2	1.24	0.0276	29	24	69	52	63	89	147	131	80	115
TRINITY_DN23808_c1_g7	XP_003398563.1	GATA-binding factor A [Bombus terrestris]	GATA(PF00320),GATA(PF00320),Androgen_recep(PF02166	5.02	-1.42	0.03	49	75	28	78	32	19	16	30	13	19
TRINITY_DN23221_c3_g5	No hits			3.98	1.3	0.03	6	8	16	8	10	21	24	24	31	21
TRINITY_DN20648_c0_g1	XP_020720068.1	paternally-expressed gene 3 protein isoform X2 [Bombus		2.79	-3.03	0.0314	9	6	6	60	16	1	2	5	0	1
TRINITY_DN22716_c3_g3	KOC69560.1	hypothetical protein WH47_05503 [Habropoda laboriosa]		4.08	-1.64	0.0365	13	60	33	18	23	10	9	10	8	9
TRINITY_DN24509_c1_g1	No hits			4.72	-1.46	0.04	32	63	61	39	21	12	6	17	20	21
TRINITY_DN23626_c1_g1	No hits			6.1	-1.1	0.04	67	126	147	105	56	40	40	50	49	52
TRINITY_DN38070_c0_g1	No hits			1.32	-2.81	0.0434	6	8	7	6	3	1	1	0	0	0
TRINITY_DN24279_c5_g5	No hits			3.92	-1.64	0.0436	12	36	31	39	12	13	6	6	8	7
TRINITY_DN19724_c0_g1	XP_003399869.1	uncharacterized protein LOC100644101 [Bombus terrestris]	DUF3731(PF12531)	5.47	-1.87	0.0462	55	180	75	91	22	10	21	28	15	40

Table S3. Identified different expression genes (DEGs) involved in nutrition between castes in the brains in *Bombus ignitus* .

Gene id	Accession number	Gene	Tag count (mean ± S.E.)		Q / W	A-value	M-value	FDR
			Queen (n=5)	Worker (n=5)				
TRINITY_DN22331_c0_g1	ICPS01081940	*VG1* (vitellogenin)	27066.4 ± 2234.1	12176.4 ±897.9	2.22	14.15	-1.15	0.0000038
TRINITY_DN22331_c0_g2	ICPS01081938	*VG2* (vitellogenin)	71 ± 25	4 ± 0.7	17.75	4.16	-3.99	0.000032
TRINITY_DN24008_c4_g2	ICPS01099189	*VG3* (vitellogenin)	477733 ± 65462.1	234972.6 ± 8011.6	2.03	18.35	-1.02	0.0003
TRINITY_DN22677_c0_g1	ICPS01087161	*TRET* (*BigTRET*) (trehalose transporter)	4112.4 ± 892.9	823.4 ± 242.1	4.99	10.85	-2.32	0.00086
TRINITY_DN22847_c2_g3	ICPS01050555	*Sugar tr* (sugar transporter)	1660 ± 102.2	814.6 ± 39.6	2.04	10.18	-1.03	0.0000055
TRINITY_DN21028_c0_g1	ICPS01076592	*RJPL* (*BigRJPL*) (royal jelly protein-like)	1274.6 ± 922.8	33.8 ± 19.3	37.71	7.71	-5.21	0.019

Table S4. Expression of genes coding enzymes involved in monoamine metabolisms and monoamine receptors in queens and workers in the brains in *Bombus ignitus* .

Monoamine	Protein type	Gene	Description	Gene id	Accession number	Tag count (mean ± S.E.)		Q / W
						Queen (n = 5)	Worker (n = 5)	
Dopamine								
	Enzyme	*BigTH*	Tyrosine hydroxylase	TRINITY_DN22889_c2_g1_i2	ICPS01051308	438.4 ± 43.4	580.6 ± 62.5	0.76
		BigDDC	Aromatic-L-amino-acid decarboxylase	TRINITY_DN22437_c0_g1_i7	ICPS01078852	21.2 ± 2.6	20.2 ± 2.9	1.05
		BigNAT	Dopamine N-acetyltransferase	TRINITY_DN22270_c0_g1_i4	ICPS01116188	223.0 ± 18.6	189.4 ± 24.6	1.18
		BigDBH	Dopamine beta-hydroxylase	TRINITY_DN23452_c4_g4_i3	ICPS01131801	307.0 ± 38.1	261.2 ± 22.6	1.18
	Transporter	*BigDAT*	Dopamine transporter (sodium-dependent	TRINITY_DN21096_c0_g1_i3	ICPS01076757	13.0 ± 1.6	12.0 ± 0.9	1.08
	Receptor	*BigDOP1*	Dopamine D1-like receptor 1	TRINITY_DN24311_c0_g1_i2	ICPS01068913	70.8 ± 2.8	68.4 ± 4.1	1.03
		BigDOP2	Dopamine D1-like receptor 2	TRINITY_DN24255_c3_g1_i3	ICPS01118583	34.8 ± 3.1	35.6 ± 3.5	0.98
		BigDOP3	Dopamine D2-like receptor	TRINITY_DN24377_c0_g6_i4	ICPS01071666	98.4 ± 7.0	77.8 ± 6.7	1.26
		BigDa-Ecd	Dopamine/Ecdysteroid receptor	TRINITY_DN23828_c4_g8_i1	ICPS01125429	1794.8 ± 119.0	2017.4 ±	0.89
Tyramine								
	Enzyme	*BigTDC*	Tyrosine decarboxylase	TRINITY_DN22747_c5_g1_i3	ICPS01009989	217.2 ± 14.8	208.4 ± 28.3	1.04
	Receptor	*BigTAR1*	Tyramine receptor 1	TRINITY_DN23415_c0_g2_i7	ICPS01130534	107.4 ± 9.4	125 ± 11.3	0.86
		BigTAR2	Tyramine receptor 2	TRINITY_DN17032_c0_g1_i1	ICPS01024365	0	0.6 ± 0.6	0.25
Octopamine								
	Enzyme	*BigTBH*	Tyramine beta hydroxylase	TRINITY_DN23452_c4_g4_i3	ICPS01131801	307.0 ± 38.1	261.2 ± 22.6	1.18
	Receptor	*BigOARb*	Octopamine receptor Oamb	TRINITY_DN24147_c0_g1_i12	ICPS01005302	153.2 ± 12.9	165.4 ± 4.4	0.93
		BigOARb1R	Octopamine receptor beta-1R-like	TRINITY_DN23538_c1_g2_i5	ICPS01025904	5.4 ± 0.7	3.4 ± 0.9	1.56
		BigOARb2R	Octopamine receptor beta-2R-like	TRINITY_DN23460_c3_g1_i1	ICPS01128989	7.0 ± 1.4	5.2 ± 1.0	1.33
		BigOARb3R	Octopamine receptor beta-3R-like	TRINITY_DN24189_c4_g1_i2	ICPS01006077	109.2 ± 8.0	95.0 ± 10.6	1.15
Serotonin								
	Enzyme	*BigTRH*	Tryptophan 5-hydroxylase	TRINITY_DN22358_c0_g1_i9	ICPS01081628	88.0 ± 15.6	103.6 ± 16.5	0.85
	Receptor	*Big5HTR1*	Serotonin receptor 1	TRINITY_DN24236_c0_g1_i19	ICPS01120149	37.0 ± 2.8	33.4 ± 4.4	1.11
		Big5HTR2a	Serotonin receptor 2alpha	TRINITY_DN24009_c1_g1_i5	ICPS01097562	670.6 ± 21.6	626.0 ± 29.0	1.07
		Big5HTR2b	Serotonin receptor 2beta	TRINITY_DN23608_c2_g2_i1	ICPS01043081	106.6 ± 8.6	120.4 ± 11.9	0.89
		Big5HTR7	Serotonin receptor 7	TRINITY_DN23819_c3_g1_i4	ICPS01124756	18.0 ± 2.4	13.0 ± 1.9	1.38

Table S5. QC results of total RNA for RNA-seq libraries.

Sample name (caste-colony-No.)	Concentration (ng / µL)	Final volume (µL)	Total amount (µg)
Queen-C6-1	469.951	46	21.618
Queen-C7-1	391.412	47	18.396
Queen-C7-2	303.413	48	14.564
Queen-C8-1	435.859	50	21.793
Queen-C8-2	392.282	49	19.222
Worker-C6-1	402.707	50	20.135
Worker-C7-1	355.404	50	17.77
Worker-C7-2	327.083	49	16.027
Worker-C8-1	279.744	47	13.148
Worker-C8-2	348.751	47	16.391

Total RNA integrity was checked by using an Agilent Technologies 2100 Bioanalyzer.

Table S6. Primer sequence of the focal genes for qPCR.

Protein type	Description	Gene id	Gene	Primer set*	Forward primer	Reverse primer
Enzyme	Tyrosine hydroxylase (TH)	TRINITY_DN22889_c2_g1_i2	*BigTH*	-	CGATCTTTGGGCTTGAAGAG	AGTTTCAAAGCGAGCATCGT
	Aromatic-L-amino-acid decarboxylase (DDC)	TRINITY_DN22437_c0_g1_i7	*BigDDC*	a	GAGTTACCTGCTCACGCACA	CCTTCGGCTGTATCAGCTTC
				b	GAGTTACCTGCTCACGCACA	TCCTTCGGCTGTATCAGCTT
	Dopamine N-acetyltransferase (NAT)	TRINITY_DN22270_c0_g1_i4	*BigNAT*	-	TGCCAACGTGAGAGCAATAG	GGTAAAGCAACGGACGGTTA
	Tyrosine decarboxylase (TDC)	TRINITY_DN22747_c5_g1_i3	*BigTDC*	-	CACGAACCACAATTTCAACG	ATCCCAACAAGTGGTTGCTC
	Tyramine beta hydroxylase (TBH)	TRINITY_DN23452_c4_g4_i3	*BigTBH*	-	TGCTATTAGCGACGATGCAC	TCCCATAATCCAGGAAGTCG
Receptor	Dopamine receptor 1 (DOP1)	TRINITY_DN24311_c0_g1_i2	*BigDOP1*	-	TAACGAAACTGCCGGATACC	CTTGCAATATGAAGCGACGA
	Dopamine receptor 2 (DOP2)	TRINITY_DN24255_c3_g1_i3	*BigDOP2*	-	GAGGGAGAAGCACGAAACTG	TGGTCGATCATTCACCAGAA
	Dopamine D2-like receptor (DOP3)	TRINITY_DN24377_c0_g6_i4	*BigDOP3*	-	CAACTACGCCCGTCGATATT	ACGCTCTATATCCGCTCGAA
Transporter	Dopamine transporter (DAT)	TRINITY_DN21096_c0_g1_i3	*BigDAT*	-	GCCGAACATTTGGGTAGAGA	GAAATGCCCTTCCACAAAGA
Others	Royal jelly protein (RJP)	TRINITY_DN21028_c0_g1_i1 (GenBank: HQ399187.1)	*BigRJPL*	a	CACGCATATTGCATTTCCAC	CAAGTTCCTCGCAAATGGAT
				b	CGTTATGGTTGTTGGTCGTG	TAATGGCAGCTTGCCTTCTT
	Insulin-like receptor	TRINITY_DN23853_c1_g1_i1	*BigILPR*	-	ATTCGGCATGGTTTACGAAG	GAGTTACCACGCCAAGAAGC
	Sugar transporter	TRINITY_DN22677_c0_g1_i3	*BigTRET*	-	GGTGCTCTCAGCGATAGGTC	CGTGACCGTATCGTTTTCCT
	Vitellogenin	TRINITY_DN20730_c0_g3_i1 (GenBank: FJ913883.1)	*BigVG*	a	CCTCTGCTCACCGAGAAAAC	CTGTTCGCGTTTCAGATTCA
				b	ACGCTGATGGTCTCGACTCT	GGCCGAGTTCACGTATTTGT
	Vitellogenin receptor	TRINITY_DN23236_c0_g1_i11	*BigVGR*	-	ACCAGTGCCTTGATCACTCC	CTTCCAAGACGAGCCTTACG
	Methopren tolelant	TRINITY_DN24405_c2_g2_i5	*BigMET*	-	CCCGACACCTCCTCTGATAA	ATCGTTTTCGTGGGTGTAGC
Reference	Actin-5C	TRINITY_DN23719_c1_g3_i9	*BigACT*	-	GATGGATGGTCCAGACTCGT	GAATCGCTGACAGAATGCAA
	40S ribosomal protein S3	TRINITY_DN23711_c0_g1_i8	*BigRPS3*	-	CAACCCGTCATGTCCTTCTT	CGCAAGTGGTATTGGTTGTG
	Glyceraldehyde-3-phosphate dehydrogenase 2 (GAPDH)	TRINITY_DN20849_c0_g1_i1	*BigGAPDH*	-	CATTCCAGCCCTTAATGGAA	CTTCAAGGGTCCTTCAGCAG

* In several genes, two primer sets (a and b) were desgined on a single gene sequence.

Table S7. Data of biogenic amine levels (pmol/protein mg) analyzed by HPLC-ECD.

Colony	Caste	Tyrosine	DOPA	Dopamine	NADA	Norepinephrine	Tyramine	Octopamine	Tryptophan	serotonin	NA5HT
Colony 1	Queen	31543.9739	53.1354325	277.23364	80.7451011	12.52222892	27.9811426	49.1097486	4050.88444	36.0675752	13.8139323
	Queen	22182.4118	57.4288516	671.031478	228.593894	9.921897793	19.5542894	56.3621018	2842.96024	17.5129504	12.9452207
	Queen	14687.3786	34.8215083	926.164225	147.997845	5.103887652	17.4926055	49.493838	2002.38531	20.7194707	11.3552581
	Queen	35986.1274	81.3231603	494.809729	127.49394	15.72129715	35.8083331	70.9474575	4573.15116	42.6949769	22.1764075
	Queen	19622.4807	48.2943944	428.787344	131.938362	9.303502453	23.8938886	58.4437191	2798.73525	37.1937913	18.3219948
	Queen	33805.2169	95.8422123	531.635837	158.659685	6.439409315	27.4141553	51.794913	4498.87288	29.7259977	17.5641172
	Queen	24594.857	45.1395993	589.738019	176.67885	6.173796147	20.1434279	55.2954372	3624.04946	23.9473575	14.5541192
	Queen	13269.0006	31.1883538	438.421959	96.2849391	4.064233384	13.3597431	36.3590826	1881.76584	17.696869	9.60183922
	Queen	16127.4968	30.8295687	369.184057	111.744297	5.745374646	14.8317724	33.2071168	2059.99057	15.1106865	9.00037176
	Queen	31803.3419	67.1883832	916.045368	214.994735	7.014166572	15.8710878	45.1499576	4002.7647	17.2644769	15.8018766
	Worker	19714.7157	39.2978221	181.094523	43.8498887	7.946094705	17.3594097	26.9587989	3254.41188	16.3164258	11.335664
	Worker	21681.2678	60.5647226	368.109952	111.498063	4.126424317	18.5866556	30.9138185	3918.60953	22.4917378	13.5109233
	Worker	24766.1305	70.4700865	645.787929	207.757951	9.317960652	21.0960327	52.0139344	2245.27453	36.5785144	16.4745343
	Worker	21299.1764	61.9527359	217.700233	77.4004989	6.120025889	21.437715	33.6895089	3769.29615	22.8508933	16.4938453
	Worker	17197.4033	53.5154925	217.667958	172.014604	8.671266099	18.4221629	41.6314349	1123.08119	24.8624052	13.7565617
	Worker	22901.1022	45.7845969	188.904815	58.1155931	7.486218389	14.8485419	28.1828105	2910.50534	16.128814	10.8625258
	Worker	19363.626	46.384478	199.461464	120.916639	2.739036955	14.8653547	44.4858017	1057.60647	21.3524178	9.88703056
	Worker	25428.4034	73.1330261	338.828418	119.389226	5.582324277	13.7539668	50.4671111	3381.37356	18.2409873	11.6788715
	Worker	11741.2188	63.0675996	115.867708	83.3068616	3.137950888	9.47004693	35.9294039	606.894333	12.9301835	7.21866257
	Worker	28210.2275	91.9160023	380.473999	124.712763	4.963274798	19.8504671	46.5549478	5033.84499	22.0143475	17.8115428
Colony 2	Queen	35043.7126	58.5067796	734.369702	113.095098	6.835923713	30.0263939	73.2679089	3892.36136	36.7677155	10.5165504
	Queen	30577.6606	58.9559909	316.172481	75.4441456	3.593470634	20.6823173	82.5156677	3696.46686	42.4772114	9.90688842
	Queen	30854.4207	72.2862587	390.999262	108.228029	6.597777036	26.3068254	87.0164871	3630.77406	54.749564	16.8246207
	Queen	29267.7388	63.0667433	682.661867	128.860918	8.665280566	20.1462257	76.7938838	3301.25139	39.3194324	9.79898429
	Queen	25021.3087	61.2715233	270.523685	68.714132	6.55221788	24.624594	90.227826	3896.63601	51.3501962	10.8517858
	Queen	22904.3773	50.4508884	345.238335	99.8295638	6.45865624	18.3168968	50.9170087	3443.60505	18.3706855	7.90503252
	Queen	23440.9342	59.7295758	845.234419	155.26015	8.432496625	33.8270562	79.5510496	4026.20684	28.7719548	11.0217995
	Queen	26864.5815	58.594842	230.288415	65.227682	3.251360634	20.9505468	50.5481625	3105.57083	19.3635053	6.32652408
	Queen	26634.5843	70.1442339	205.914054	70.340199	5.644599057	21.7431207	52.9906441	2865.07566	19.4898393	7.04254746
	Queen	18674.5779	33.4794269	490.561349	92.7644148	5.072761289	20.2935543	44.3269789	2706.42395	19.9859073	9.16545866
	Worker	14103.6934	31.7367948	154.417387	42.4277842	3.018064122	13.6907324	37.7357056	1828.95469	21.1758572	5.47936227
	Worker	26032.8595	63.4824633	180.272394	47.4890339	5.76170087	28.3799884	83.0890226	2982.37477	48.323886	10.2628764
	Worker	16307.2118	39.9494092	108.048768	33.5719495	1.830734064	14.5938061	58.2583259	2507.70643	33.3159245	8.35029994
	Worker	16449.3492	52.2991757	409.4346	114.964234	0.44067205	22.819101	69.5397749	2118.67563	40.6780296	9.66602398
	Worker	21263.2432	88.9920189	280.464259	212.186208	0.936992723	22.5623869	104.248301	1358.53989	62.0591636	18.0795109
	Worker	30551.8621	86.2851332	231.590998	155.95851	0.274353951	25.9597265	65.7608963	2726.1212	21.9449527	13.0091452
	Worker	11916.018	62.4037787	257.007176	178.62371	0.326665202	17.709278	59.0518826	752.870604	20.4814761	9.43687451
	Worker	24481.883	62.5173351	348.138164	110.791815	5.901871688	21.0540626	57.8665027	2623.67581	18.7836433	4.94849249
	Worker	23537.2556	65.1712758	307.273555	93.6163947	4.76432719	19.5358807	51.1915918	3235.90798	17.5718241	6.30804032
	Worker	23211.6994	64.1189937	273.622544	86.0409882	3.046537324	14.3309888	52.6123387	3231.3385	17.7555177	6.62648384

(pmol/protein mg)

Table S8. Data of relative gene expression levels analyzed by qPCR.

Colony	Caste	*BigTH*	*BigDDC*	*BigNAT*	*BigTDC*	*BigTBH*	*BigDOP1*	*BigDOP2*	*BigDOP3*	*BigDAT*	*BigVG-a*	*BigVG-b*	*BigVGR*	*BigRJPL-a*	*BigRJPL-b*	*BigILPR*	*BigTRET*	*BigMET*
Colony 3	Queen	1.42449	0.95587	1.17343	0.75835	0.94699	1.03665	0.75241	1.61796	0.95503	0.06066	0.01935	0.08937	0.621	0.777694	1.96322	2.19653	1.41474
	Queen	1.30374	1.28947	1.56759	1.0304	1.08434	1.24619	0.76426	1.77173	1.17514	0.49	0.14212	0.10283	1.681038	1.420233	1.99031	3.21394	0.89342
	Queen	1.55602	1.28719	2.04092	0.82925	0.95492	1.84164	0.82784	1.70263	1.23359	0.16079	0.0455	0.10905	1.476474	1.634556	2.37752	2.56221	1.31706
	Queen	0.86155	0.94558	1.25202	0.67638	0.91407	1.23062	0.78775	1.1753	0.78892	0.45778	0.27522	0.07251	0.755697	0.789218	1.15359	4.151	0.63281
	Queen	1.40935	0.71184	0.9321	0.72777	0.73592	0.98901	0.79555	1.34655	0.69905	0.43975	0.24385	0.03075	1.031112	1.709644	0.81733	2.51661	0.47328
Colony 4	Queen	0.4355	0.85665	1.97683	0.50914	0.99215	1.08589	0.67827	0.87342	0.69798	125.851	136.487	2.50795	49.77558	141.3622	1.16753	11.358	0.66559
	Queen	0.53396	0.72721	1.50707	0.53362	0.69686	1.11367	0.63991	1.07761	0.7486	13.0512	11.7155	1.6976	11.67778	26.79842	1.53321	6.04439	0.75597
	Queen	0.53363	0.77352	1.33692	0.50194	0.78506	1.13705	0.62829	0.88835	0.62676	15.5419	15.4044	2.31004	1.608654	2.309384	1.04909	6.50918	0.64809
Colony 5	Queen	0.64819	0.80044	1.1154	0.4852	0.74035	0.93417	0.56654	0.87372	0.62032	0.38363	0.2133	0.06303	0.969791	1.16433	0.94169	2.53004	0.52363
	Queen	0.68802	0.83737	1.3067	0.64505	0.86579	1.12485	0.65088	1.08229	0.72742	4.39246	3.55966	0.07962	6.241467	11.10443	1.12678	9.049	0.60676
	Queen	1.17531	1.20922	1.21441	0.63958	0.96369	1.06695	0.66932	1.22612	0.82705	0.42329	0.22318	0.08124	1.101684	1.249334	1.40525	1.99155	0.75567
Colony 3	Worker	1.18162	1.17431	0.95847	0.89521	1.20399	1.15547	0.99003	1.40734	1.07244	2.07298	1.84297	0.10174	1.062475	1.473685	1.05595	1.70897	0.67991
	Worker	1.52877	1.49646	0.87417	0.77187	1.22291	1.10524	0.76723	1.53125	1.41685	3.87259	2.15156	0.08907	3.127531	4.61598	1.23289	1.21602	0.67671
	Worker	1.16335	1.43716	1.64539	0.62807	1.25374	1.76667	0.75501	1.61592	1.39349	2.05039	0.78054	0.12228	1.08731	1.243649	1.63942	1.28555	0.83915
	Worker	1.30406	0.96096	1.12944	0.57293	0.77828	1.09551	0.69545	1.26123	0.96242	1.70329	0.9717	0.09999	0.740519	0.689418	0.80429	1.2589	0.54184
	Worker	1.94762	0.81318	0.48157	0.49077	0.92359	0.91333	0.90879	1.62956	0.83382	0.76069	0.52471	0.04228	0.641087	1.15244	0.67978	0.74372	0.42661
Colony 4	Worker	0.82295	0.91088	1.47063	0.275	0.91661	0.9365	0.83753	1.18344	0.96563	0.44325	0.22564	2.40622	0.719175	0.807327	1.03207	0.56411	0.64981
	Worker	0.72708	0.77466	1.07796	0.43695	0.63302	0.92987	0.55421	1.21046	1.01849	16.2662	16.4791	2.15901	1.232489	1.542266	0.5343	1.60498	0.51014
	Worker	0.9847	0.80075	1.18725	0.31393	0.50015	1.10864	0.66244	1.34859	0.99046	0.52892	0.33315	1.88862	0.59481	0.553417	0.81693	0.74559	0.58532
Colony 5	Worker	0.97239	0.7921	0.95503	0.37604	0.67592	0.97959	0.67292	1.16761	0.96834	0.11142	0.05935	0.04192	0.456275	0.452871	0.82658	0.63781	0.48399
	Worker	1.13445	0.88211	0.98681	0.436	0.72029	0.98421	0.6792	1.36685	1.09168	2.46395	2.02847	0.0672	1.04113	1.245999	1.12322	0.89199	0.60261
	Worker	1.16712	1.03092	0.98448	0.57018	0.90693	1.04626	0.7352	1.32505	0.97456	1.07121	0.70659	0.05055	0.752222	0.760439	0.64291	1.03569	0.56997
